# Grandparenting and Well-Being of Hong Kong Older Adults: The Mediating Role of Intergenerational Relationships

**DOI:** 10.1093/geroni/igaa057.1193

**Published:** 2020-12-16

**Authors:** Daniel W L Lai, Jessica J Li, Vincent Lee

**Affiliations:** The Hong Kong Polytechnic University, Kowloon, China

## Abstract

Grandparenting and intergenerational relationship play important roles in some older adults‘ later life, especially older people of Chinese culture. This study investigated the relationship between grandparenting activities, intergenerational relationship, and psychosocial well-being of Hong Kong Chinese older adults. A representative sample of 507 grandparents (aged 55+) were telephone surveyed in June to July 2019. Level of involvement in grandparenting activities was measured. Resilience and happiness were measured by Connor-Davidson Resilience Scale and Subjective Happiness Scale. Two single-item instruments were adapted to capture the relationships between older adults and adult children, and between grandparents and grandchildren, respectively. A series of linear regressions and mediation tests with bootstrap approach were performed to examine the relationships between grandparenting activities, intergenerational relationship, and resilience and happiness. After controlling for socio-demographics, the frequency of grandparenting activities correlated positively with resilience and happiness. The relationship was partially mediated by inter-generational relationships including the relationships with adult children and grandchildren. The findings have concluded that grandparenting involvement and satisfactory intergenerational relationship are protective factors of health and wellbeing. Future healthy aging policy-making or programming should expand the scope from focusing on individual older adults to strategies of achieving the family-friendly goal so that intergenerational relationships could be better nurtured, benefiting not just the family as a functional unit but also the older adults’ healthy aging.

